# Synthesis, Molecular Docking and Anticancer Activity of Diflunisal Derivatives as Cyclooxygenase Enzyme Inhibitors

**DOI:** 10.3390/molecules23081969

**Published:** 2018-08-06

**Authors:** Göknil Pelin Coşkun, Teodora Djikic, Taha Bartu Hayal, Nezaket Türkel, Kemal Yelekçi, Fikrettin Şahin, Ş. Güniz Küçükgüzel

**Affiliations:** 1 Department of Pharmaceutical Chemistry, Faculty of Pharmacy, Cumhuriyet University, Sivas 58140, Turkey; goknilpelincoskun@gmail.com; 2 Department of Bioinformatics and Genetic, Faculty of Engineering and Natural Sciences, Kadir Has University, Istanbul 34083, Turkey; teodorica.djikic@gmail.com (T.D.); kyelekci@gmail.com (K.Y.); 3 Department of Genetics and Bioengineering, Faculty of Engineering and Architecture, Yeditepe University, Kayışdağı, Istanbul 34755, Turkey; taha.bartu.hayal@gmail.com (T.B.H.); nezaket.turkel@gmail.com (N.T.); fsahin@yeditepe.edu.tr (F.Ş.); 4 Department of Pharmaceutical Chemistry, Faculty of Pharmacy, Marmara University, Istanbul 34668, Turkey

**Keywords:** diflunisal, thiosemicarbazide, 1,2,4-triazole-3-thione, anticancer, COX-2, docking

## Abstract

Cyclooxygenase enzymes play a vital role in inflammatory pathways in the human body. Apart from their relation with inflammation, the additional involvement of COX-2 enzyme with cancer activity was recently discovered. In some cancer types the level of COX-2 enzyme is increased indicating that this enzyme could be a suitable target for cancer therapy. Based on these findings, we have synthesized some new diflunisal thiosemicarbazides and 1,2,4-triazoles and tested them against androgen-independent prostate adenocarcinoma (PC-3), colon carcinoma (HCT-116), human breast cancer (T47D), breast carcinoma (MCF7) and human embryonic kidney (HEK-293) cell lines. Specifically, the diflunisal and thiosemicarbazide functionality are combined during the synthesis of original compounds anticipating a potency enhancement. Compounds **6**, **10**, **15** and **16** did not show cytotoxic effects for the HEK293 cell line. Among them, compounds **15** and **16** demonstrated anticancer activity for the breast cancer cell line T47D, whereas compounds **6** and **10** which are thiosemicarbazide derivatives displayed anti-tumourigenic activity against the PC-3 cell line, consistent with the literature. However, no activity was observed for the HCT-116 cancer cell line with the tested thiosemicarbazide derivatives. Only compound **16** displayed activity against the HCT-116 cell line. Therefore, it was speculated that the diflunisal and thiosemicarbazide functionalities potentiate anticancer activity on prostate cancer and the thiosemicarbazide functionality decreases the anticancer activity of diflunisal on colon cancer cell lines. In order to gain insight into the anticancer activity and COX-2 inhibition, molecular docking studies were carried out for COX-1 and COX-2 enzymes utilizing the newly synthesized compounds **15**, and **16**. Both **15** and **16** showed high selectivity and affinity toward COX-2 isozyme over COX-1, which is in agreement with the experimental results.

## 1. Introduction

Cyclooxygenase (COX) enzymes play an important role in the synthesis of prostaglandins. So far, three isoenzymes of COX have been discovered; COX-1, COX-2 and COX-3. The constitutive COX-1 isoenzyme which can be found throughout the body, where it is responsible for the protection of the gastrointestinal track, whereas COX-2 is an inducible isoenzyme responsible for inflammation [1,2]. COX-3 enzyme is a variation of COX-1, encoded by the same gene [3]. Recently, it was suggested that COX-3 is the main target of paracetamol [4]. The important discovery of the role of COX-2 isoenzyme, led researchers to investigate the potential involvement of these enzymes in pain [5]. Recent studies have been carried out to obtain some selectivity between COX-1 and COX-2 enzymes. Since COX-1 isoenzyme is responsible for the protection of the gastrointestinal system and COX-2 is responsible for pain, the selective inhibition of COX-2 is extremely important. Therefore, development of non-steroid anti-inflammatory drugs (NSAIDs), has received the attention of researchers seeking to find potent and selective COX-2 inhibitors [6]. Up-regulated expression of COX-2 isoenzyme under pathological conditions was proved to be involved in inflammation and various cancers types. The COX-2 is commonly over-expressed in several cancers including esophageal, gastric, pancreatic, colorectal and prostate cancers [7]. Thus, studies on the anti-cancer effects of COX-2 selective NSAIDs drugs have opened the ways for new research areas. [8]. Due to the noxious side-effects of various anti-cancer agents on the market, it is important to develop potent anti-cancer agents with less adverse effects.

Diflunisal (2′,4′-difluoro-4-hydroxybiphenyl-3-carboxylic acid), a salicylic acid derivative, is known as a NSAID. It is a selective inhibitor of COX-2 isoenzyme over COX-1 [9]. Diflunisal and its derivatives have been reported to possess diverse biological effects, including anti-cancer activities [10,11,12,13,14,15]. In order to increase the suppressive effect of diflunisal on the proliferation of cancer cells, some diflunisal derivatives were synthesized by adding new functional groups to the diflunisal scaffold. For this purpose, medicinal chemists use different tools including fragment-based drug design to optimize the potency, unwanted property and selectivity of a given lead drug structure toward a given targeted cancer cells. The obtained structures were tested to check their biological activities for cancer cell lines. Thiosemicarbazides, which are intermediate products of the synthesis of many biological active compounds, have attracted the attention of researchers due to of their clinical use and diverse biological activities [16]. 1,2,4-Triazole-3-thiones were reported to possess different biological activities [17]. In addition, Süzgün et al. reported that etodolac triazole possesses anticancer effect [18].

Some of the known anti-cancer agents are not very effective, as many of them have inadequate ADMET properties and easily induce resistance. There is also a considerable diversity in resistance of various cells within the same tumor. Thus, development of safe and effective new agents with less side effects and reduced drug resistance for long-term treatment of cancer is highly required [19]. Diflunisal and diflunisal derivatives are associated with cancer prevention [15,20,21]. There is a meaningful correlation between the degree of their COX-2 selectivity and anti-cancer activity [22]. The mechanism underlying the chemo-preventative actions of NSAIDs are not clear, though suppression of prostaglandin E2 synthesis, particularly via inhibition of cyclooxygenase (COX)-2 activity, has been suggested to be important reason [23]. On the other hand, selective COX-2 inhibitors have been reported to have serious cardiovascular adverse effects, so their usage is limited for chemo-prevention of cancer [24]. Therefore, we have mainly focused on developing more selective and potent anti-cancer agents with less adverse effects and decreased drug resistance.

In this study, we hypothesized that the conversion of carboxylic acid group of diflunisal into thiosemicarbazides and 1,2,4-triazole-3-thiones would enhance the selectivity of the drug towards cancer cells. Accordingly, we have some synthesized new thiosemicarbazides and 1,2,4-triazole-3-thiones of diflunisal and elucidated their structures by FT-IR, ^1^H-NMR and ^13^C-NMR. Their purity was proven by TLC, HPLC and elemental analysis. We have also investigated their anticancer activity on PC-3, HCT-116, T47D and MCF-7 cancer cell lines using cell proliferation assays. Among the tested compounds; compounds **6**, **10**, **15** and **16** showed anticancer activity against prostate adenocarcinoma (PC-3), colon carcinoma (HCT-116), and human breast cancer (T47D) cancer cell lines. None of the tested compounds were found active against the MCF-7 cell line. In order to correlate the anticancer activity and selectivity with COX-2 inhibition, molecular docking studies were performed utilizing both COX-1 and COX-2 as target enzymes with the newly synthesized compounds. These in silico studies, suggest that compounds **15** and **16** show high selectivity toward COX-2 isoenzyme over COX-1, agreeing with the experimental results.

## 2. Results and Discussion

### 2.1. Chemistry

Diflunisal (2′,4′-difluoro-4-hydroxybiphenyl-3-carboxylic acid) was converted into its corresponding diflunisal ester (**1**) and diflunisal hydrazide (**2**) according to a reported procedure [10]. In brief, diflunisal hydrazide was converted into substituted thiosemicarbazides with overall yields ranging between 65–93% by treatment with suitable aryl isothiocyanates in ethanol. Different types of solvents were used in the thiosemicarbazide preparation such as dioxane, acetonitrile, benzene, methanol, tetrahydrofuran, isopropyl, pyridine, however, the solvent of choice was ethanol, as it is safe and easy to use in synthesis. These reactions were all monitored by TLC (mobile phase: petroleum benzene/ethyl acetate 50:50, *v*/*v*). After two crystallizations from ethanol, the purities of the compounds were checked by HPLC.

Compounds **3**, **4**, and **5** were reported in our previously study [25]. The structures of the newly synthesized thiosemicarbazides **6**–**10** were confirmed by FT-IR, ^1^H-NMR, ^13^C-NMR and HR-MS. The results gained from the spectral data were all compatible with the proposed structures. The FT-IR data showed that hydrazide C=O stretching bands had shifted from 1641 cm^−1^ to 1622–1651 cm^−1^ which confirmed the formation of an amide functionality. Further, C=S stretching bands were detected between 1238–1274 cm^−1^, which also proved the presence of characteristic thiosemicarbazide C=S stretching bands. The ^1^H-NMR spectra showed the chemical shifts of the N_1_, N_2_ and N_4_ protons which also confirmed the formation of the thiosemicarbazide functionality. The thiosemicarbazide protons were observed between 13.71–11.99; 12.03–10.65 and 8.17–11.96 ppm, respectively. Formation of thiosemicarbazides was also confirmed by ^13^C-NMR studies. The presence of a thiosemicarbazide functionality produces certain C=O and C=S peaks. In this study, these C=O and C=S peaks were observed between 166.00–169.00 and 180 ppm, respectively. For further evaluation, high resolution mass spectrometry (HR-MS) was performed for three thiosemicarbazide derivatives (compounds **4**, **6** and **9**). These HR-MS studies confirmed the molecular weights and empirical formula within 8 mmu. The molecular ion peaks were observed as expected. Mass fragments of the compounds also supported the expected structures. The main fragment of the two compounds formed by the loss of isothiocyanate part of the compound, which was detected as [M-RNCS]^+^. Beside the main fragment, OH radical and halogen losses were detected in both compounds.

**Figure 1:**
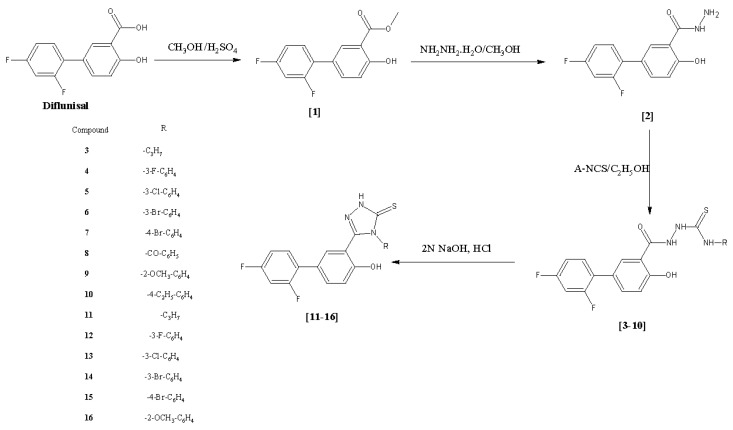
Synthetic route to newly synthesized diflunisal thiosemicarbazides (**3**–**10**) and 1,2,4-triazole-3-thiones **11**–**16**.

In order to prepare the 1,2,4-triazole-3-thione derivatives **11**–**16**, ring closures were carried out with 2 N NaOH for 4 h using the reported method [18]. Adding one more step for triazole compounds instead of trying to synthesize them from hydrazides was safer and improved the overall yield (65–98%). However, attempts to cyclize compounds **8** and **10** failed. There are not many different methods for the synthesis of 1,2,4-triazole-3-thiones except for synthesizing them from corresponding thiosemicarbazides. We tried using 2% NaOH and 4 N NaOH medium instead of 2 N NaOH for the the cyclization but all three methods failed. Diflunisal 1,2,4-triazole-3-thione derivatives **11**–**16**, were neutralized with concentrated hydrochloric acid after monitoring the reaction with TLC. The purity of the compounds were confirmed with both TLC and HPLC as performed for the thiosemicarbazides using the same methods. FT-IR spectra of the newly synthesized diflunisal triazoles supported the cyclization. The disappearance of the amide C=O and formation of C=N stretching bands proved the triazole synthesis. The C=N and C=S stretching bands of the 1,2,4-triazole moiety were detected between 1620–1633 cm^−1^ and 1240–1269 cm^−1^, respectively. The C=S stretching bands also confirmed that all the synthesized 1,2,4-triazoles are in their thione form as no stretching bands corresponding to –SH groups were observed between 2550–2600 cm^−1^. ^1^H-NMR spectral data also confirmed the formation of triazole compounds. The N_1_, N_2_ and N_4_ protons of the thiosemicarbazide were not detected in the 1,2,4-triazole compounds. Instead, N-H peaks of 1,2,4-triazole were observed between 13.76–14.16 ppm. There was no peak between 1.0–3.7 ppm which might be attributed to a -SH function and this finding also supported the presence of the thione form of the 1,2,4-triazole compounds **11**–**16**. All the other aromatic protons of the compounds were observed in the expected regions. In ^13^C-NMR studies, all the carbons were observed in the expected regions. The C=N and C=S carbons that belong to triazole functionality were observed between 155.47–155.75 and 167.58–168.19 ppm, respectively. No carbon peaks of thiosemicarbazide C=O and C=S were observed; and this was accepted as a proof of the synthesis of 1,2,4-triazoles. In ^13^C-NMR studies, the structure of compounds was confirmed to present in thione form with the observation of C=S peaks. HR-MS studies were performed on two selected triazole compounds, compounds **14** and **16**. HR-MS studies confirmed the molecular weights and empirical formulas within 8 mmu. The molecular ion peaks were observed as expected. The anticipated compound fragments were observed in the mass spectra. The main observed fragments of the two compounds were their [M-S]^+^, [M-OH]^+^ and [M-RNCS]^+^ peaks (Figure 1).

### 2.2. Biological Evaluation

#### 2.2.1. Anticancer Activity

A number of studies have reported the association between COX-2 overexpression and cancer progression. Expression of the COX-2 isoenzyme is upregulated in many human cancers, including gastric, breast, lung, colon, esophageal, prostate and hepatocellular carcinomas [26]. It has been shown that diflunisal selectively inhibits the COX-2 enzyme [15]. Furthermore, diflunisal derivatives have been reported to possess anticancer activity against leukemia [14,20] and hepatocellular [14] cancer cell lines. In the relevant studies with diflunisal, thiosemicarbazide and 1,2,4-triazole-3-thione functionalities were correlated with anticancer activity [22,27,28,29,30,31,32]. Therefore, in this study, we selected to study diflunisal thiosemicarbazides **3**–**10** and diflunisal 1,2,4-triazole-3-thiones **11**–**16** to investigate their cytotoxic effect against to cancer cells. Four different cancer cell lines, namely, prostate adenocarcinoma (PC-3), colon carcinoma (HCT-116), human breast cancer (T47D), breast carcinoma (MCF7) and a normal human embryonic kidney cell line (HEK293) were used to evaluate the anticancer activity of newly synthesized compounds.

Firstly, the half maximal inhibitory concentration (IC_50_) values of all the synthesized compounds were determined for all the cell lines used in this study by a MTS assay. Compounds for which the IC_50_ value against HEK293 cell line was twice as high as the IC_50_ value for at least one cancer cell line (compound **6**, **10**, **15**, **16**) were considered active (Table 1) and included into structure-activity analysis. 

Further experiments were performed by using selected active compounds. There are reliable studies about the anti-colon cancer activity of diflunisal and the anti-prostate cancer activity of the thiosemicarbazide functionality [22]. Therefore, here prostate cancer (PC-3) and colon cancer (HCT-116) cell lines were selected to study the anticancer activity of diflunisal and thiosemicarbazide. We combined the diflunisal and thiosemicarbazide functionalities in order to increase the anticancer activity of the resulting compounds. The expected results were obtained for the PC-3 cancer cell line (the compounds showed activity, with IC_50_ values as 41.8 µM for compound **6** and 11.7 µM for compound **10**), however, no activity was observed for the HCT-116 cancer cell line with the thiosemicarbazide derivatives. Therefore, we assumed that diflunisal and thiosemicarbazide functionality enhance anti-prostate cancer activity but the thiosemicarbazide functionality decreases the activity of diflunisal on colon cancer cell lines [15]. Comparing the differences between the active thiosemicarbazide molecules, alkyl substitution increased the activity; whereas halogen substitution showed more decreased activity than compound **10**.

Similar to the anticancer activities of thiosemicarbazides, 1,2,4-triazole-3-thiones were reported to have diverse biological activities, including anticancer activity [17]. There are several studies reporting the activity of the triazole functionality against breast cancer. In fact, letrozole and anastrozole are anticancer drugs (aromatase inhibitors) in breast cancer treatment on the market. In this study, we used the MCF7 and T47D breast cancer cell lines. Both cancer cell lines were isolated from tumours located in breast, but they differ from each other in a mutation in the p53 gene. The wild-type form of the p53 gene is expressed in the MCF7 cancer cell line, whereas in the T47D cancer cell line the p53 gene is mutated [33]. Among the tested compounds, compounds **15** and **16** which possess a triazole functionality showed activity against the T47D cell line, whereas for the MCF7 cancer cell line none of the compounds displayed any activity. These contrasting effects could be related to differential p53 expression levels. The presence of the wild-type form of p53 in the MCF7 cell line may promote its resistance against all of the compounds tested in this study. Furthermore, it is well established in the literature that the MCF7 cell line has multidrug resistance compared to the T47D cell line because of excessive expression of P-glycoprotein drug transporter gene and having an estrogen receptor subunit alpha rather than estrogen receptor subunit beta, unlike T47D [34,35,36]. It can be deduced from the results that compounds bearing a methoxy substituent are more important for T47D activity than halogen substituted ones. A compound bearing bromo substitution showed activity, with an IC_50_ value of 43.4 µM (compound **15**) and a compound bearing methoxy substitution showed activity with an IC_50_ value of 27.3 µM (compound **16**) for the T47D cancer cell line. Thus, it can be concluded that electron donating groups increase the anticancer activity on the T47D cancer cell line. When comparing the activity of these triazole compounds on other cancer cell lines, compound **16** showed activity against the HCT116 cancer cell line with an IC_50_ value of 6.2 µM and compound **15** did not show any activity to the other cancer lines (PC-3 and HCT116). The differences between these results could be related with the cancer cell types. The HCT116 cell line is known to have K-RAS activation and PIK3CA mutation [37]. Because of that, this cancer cell line is resistant to a number of anticancer drugs and even epidermal growth factor inhibitors [38,39]. The PC-3 cancer cell line differs from other prostate cancer cell lines in its synthesis of androgen receptor (AR) and prostate specific antigen (PSA), indicating that survival does not depend on androgen [40]. Furthermore, PC-3 is a CD44 positive cancer cell line and therefore, it is thought to have stem cell features [41]. Like the HCT116 cancer cell line, PC-3 is also resistant to many anticancer drugs. However, we still found immensely significant activity from compound **16** against HCT116 cancer cell line. In addition to that, the triazole functionality did not possess any activity against the PC-3 cancer cell line., and theIC_50_ values of the PC-3-active compounds demonstrated that the thiosemicarbazide functionality is more significant for this cell line.

In conclusion, the comparison of the compounds **6** and **10** indicates that alkyl substitution increases the anticancer activity on the PC-3 cell line. On the other hand, only bromophenyl derivatives showed activity in this study; which proved the importance of the bromine atom instead of chlorine or fluorine. Compound **16** has a triazole functionality and has methoxyphenyl substitution. Taking everything said into account, regarding the activity against colon cancer, small molecules are fundamental and electron donating groups are central for the anticancer activity. Compounds **15** and **16** showed the best activity on the T47D breast cancer cell line as these compounds have triazole functionalities. When the triazole is replaced by a thiosemicarbazide, the activity decreases as was observed in compound **6**. For the anticancer activity against breast cancer line, the triazole functionality has significant importance compared to the thiosemicarbazide one.

#### 2.2.2. Effect of the Synthesized Compounds on DNA Synthesis

**Figure 2:**
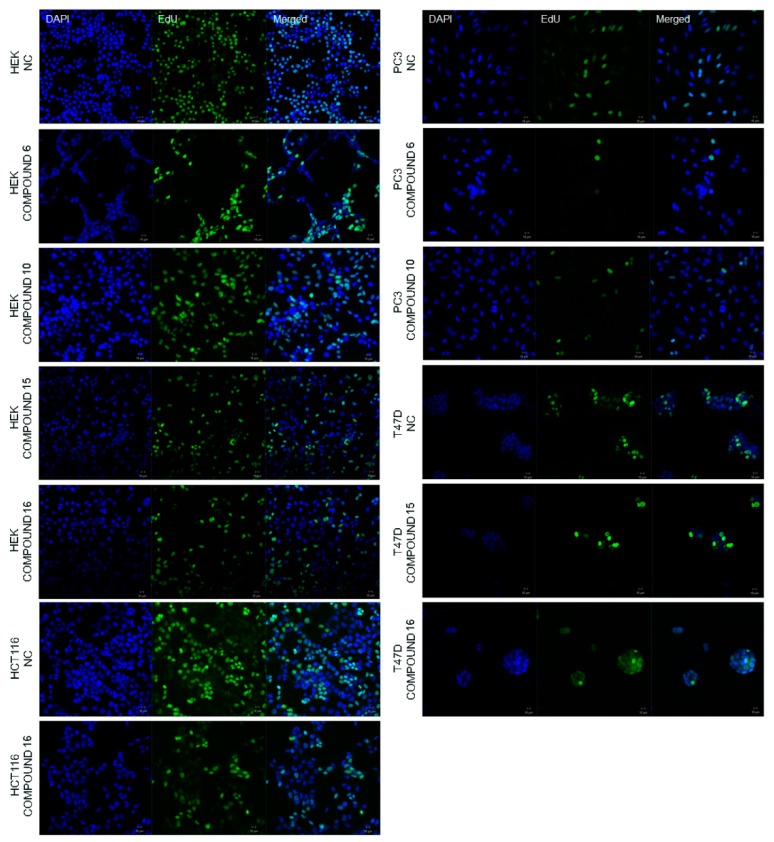
DAPI and EdU staining patterns of HEK293, PC-3, HCT116 and T47D cell lines. DAPI (in blue, left panel) shows all the cells, EdU shows newly synthesized DNA (in green, middle panel and merged image, right panel). As shown, when treated with compounds, the rate of DNA synthesis decreased compared to the negative control group of each cell line individually, except for the non-cancer cell line HEK293. All images captured at 20× magnification.

Determination of IC_50_ values of the compounds was subsequently followed by observation of newly synthesized DNA in the cells. It is well-known that DNA synthesis is correlated with cell proliferation [44] and 5-ethynyl-2′-deoxyuridine (EdU) staining enables one to visualize newly synthesized DNA to determine cell proliferation. EdU is a thymidine analogue that integrates DNA during DNA synthesis, therefore here it stained only newly synthesized DNA during the experimental procedure (in green fluorescence, Figure 2). 4′,6-Diamidino-2-phenylindole (DAPI) stain binds strongly to A-T rich areas in DNA and passes through cell membranes easily, thereby it stains nucleus of the all cells regardless of whether the DNA is newly synthesized or not (in blue, Figure 2). It was revealed that COX-2 enzyme inhibition via the newly synthesized compounds decreases the rate of DNA synthesis in the HCT116, PC-3 and T47D cell lines, whereas the DNA synthesis in HEK293 cells did not appear to be affected by the treatment of any of the compounds found active based on IC_50_ values (Figure 2). These results indicated that the rate of DNA synthesis decreased dramatically in the presence of our newly synthesized compounds in cancer cell lines compared to the healthy cell line HEK293.

#### 2.2.3. Effect of Synthesized Compounds on Apoptosis

**Figure 3:**
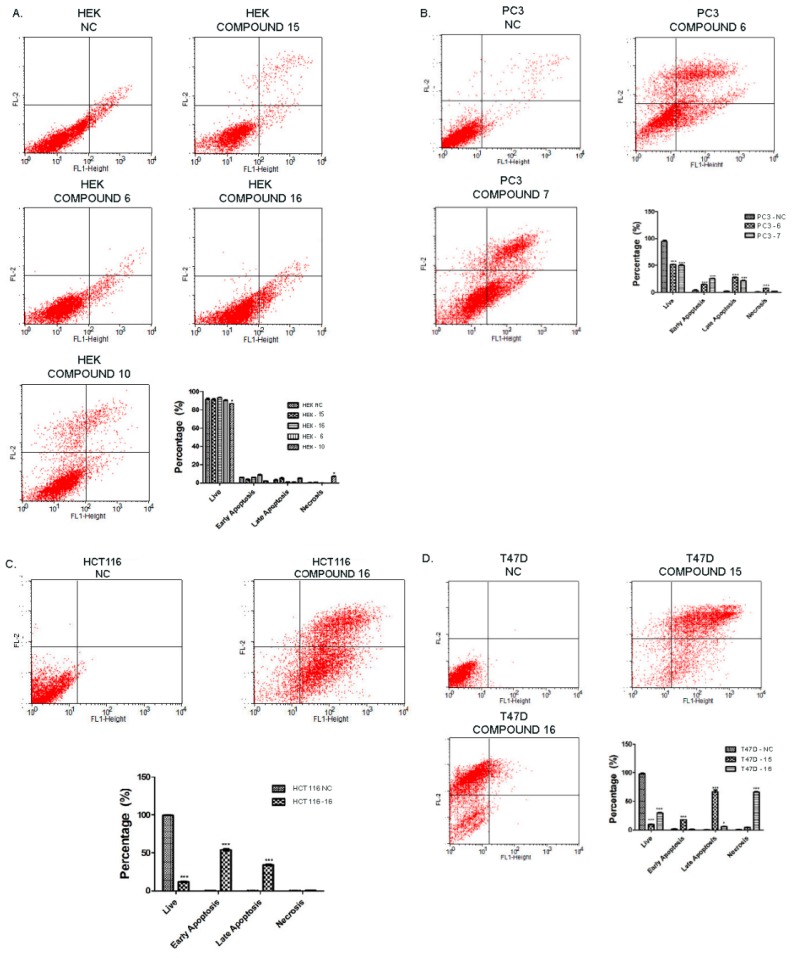
Determination of apoptosis and necrosis potential of synthesized compounds on HEK293, PC-3, HCT116 and T47D cell lines. (**A**) Compounds tested on HEK293 cell line did not exhibit any cytotoxic effects, except compound **10** with a slight insignificant increase in necrosis; (**B**) Treatment of PC-3 cells with compounds **6** and **10** resulted in increase of late apoptosis markers; (**C**) Compound **16** displayed extensive apoptotic effects (both early and late markers were elevated) on the HCT116 cancer cell line; (**D**) T47D cancer cells underwent vast late apoptosis through the treatment with compound **15**, whereas compound **16** caused necrosis of T47D cancer cells (* *p* < 0.05, ** *p* < 0.01, *** *p* < 0.001).

Evaluation of the effect of the synthesized compounds on apoptosis was determined by the FITCH-AnnexinV/PI protocol. Damaged cell membrane of necrotic cell enables propidium iodide (PI) to bind DNA and induces a red fluorescence, although it is excluded by non-damaged cells, select live and apoptotic cells. FITCH-Annexin V has the ability to bind to phosphatidylserine which is a specific marker of apoptosis. Figure 3 demonstrates the results of FITCH-Annexin V/PI of PC-3, T47D, HCT116 and HEK293 cell lines after treatment with the COX-2 inhibitors synthesized in this study. The lower left panel of the quadrant of the cytograms indicates live cells due to exclusion of PI and non-bound Annexin V.

The lower right quadrant shows early apoptotic cells and the upper right quadrant shows late apoptotic cells by virtue of no PI inclusion but positive Annexin V binding. The upper left panel of the cytogram represents necrotic cells which are positive for PI. Each cytogram of each cell was analyzed and a histogram graph was drawn for further visual information. Overall, treatment of HEK293 with active compounds (compounds **6**, **10**, **15**, **16**) did not result in apoptosis (Figure 3A). Although slightly more necrotic and late apoptotic cells were observed with the treatment of HEK293 with compound **10**, it was not significant when compared to its effect on PC-3 cancer cells. PC-3 cancer cells undergo apoptosis highly with the treatment of compound **6** and compound **10** when it is compared to non-treated negative control (Figure 3B). Compound **16** with a triazole functionality had an extensive apoptotic effect on HCT116 cancer cell line (Figure 3C). Most of the HCT116 cells went through early and late apoptosis when treated with this compound (Figure 3C), whereas it resulted in necrosis in the T47D cancer cell line. Furthermore, compound **15** led to cell death in T47D cell line largely through late apoptosis following early apoptosis indicating that these cells went through programmed cell death quite rapidly and thereby started to lose their cell membrane integrity. To sum up, all the compounds that are tested against to different cancer cell lines that were found to be active according to their IC_50_ values demonstrated higher cytotoxicity in tumor cells than normal cells (HEK293).

#### 2.2.4. Docking Studies

In an attempt to explain the biological activity and the difference in selectivity profiles of the newly synthesized 1,2,4-triazole-3-thiones toward COX subtypes based on their orientation and binding patterns, the molecules were docked into the active sites of COX-1 and COX-2, respectively, using AutoDock 4.2 [45].

**Figure 4:**
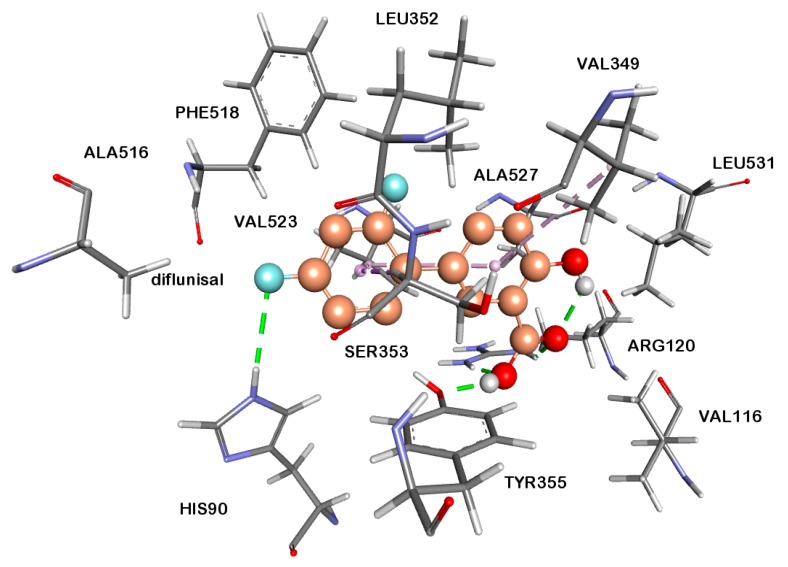
3D representation of predicted binding mode and protein-ligand interactions of diflunisal to COX-2 enzyme. H-bonds occur between hydroxyl groups of hydroxybenzoic acid part of diflunisal and TYR355 and ARG120 amino acid side chains and HIS90 monocation with the fluoro substituent of the fluorophenyl ring.

**Figure 5:**
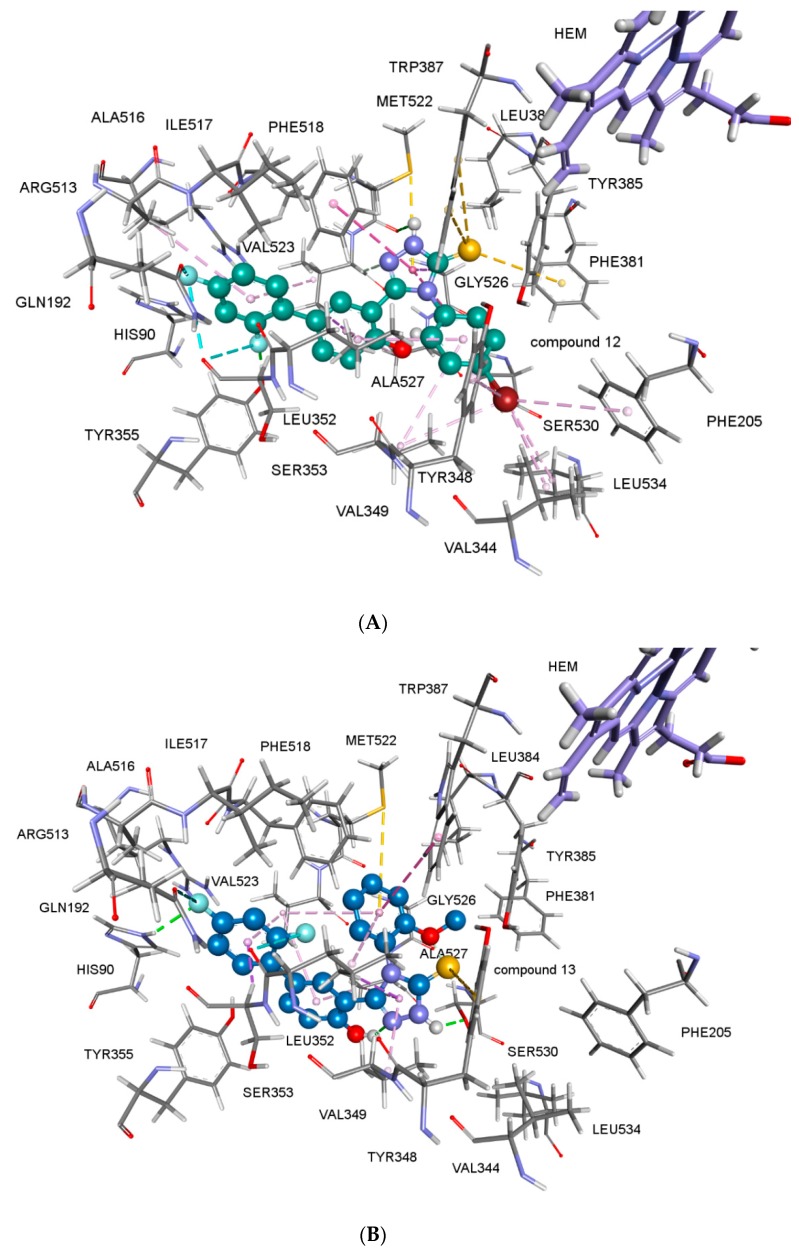
3D representations of the predicted binding mode and protein-ligand interactions of compounds **15** (**A**) and **16** (**B**) for COX-2. Halogen interactions of fluoro substituents with LEU352 are shown in blue dashed lines; hydrophobic interactions of aromatic rings with side chains of Phe205, Val344, Tyr348, Phe381, Tyr355, Trp387, Ala527 and Leu534 are shown in pink. Two hydrogen bonds of compound **16** (**B**): HIS90 monocation with fluoro substituent, and the hydroxyl group of SER530 and the triazole ring are represented with green dashed lines. Yellow lines represent S/π interactions of Met522 and with the aromatic rings of compounds. Compound **15** (**A**) showed one more S/π interaction between the sulphur of the 1,2,4-triazole-3-thion ring with the aromatic ring of Phe381.

Most of the compounds were found to be potential inhibitors of both COX-1 and COX-2 enzymes (Table 2). As expected, all of the tested compounds showed more affinity to COX-2, rather than COX-1 enzyme. The COX-2 enzyme activities of the compounds correlate their anticancer activity results. According to the docking studies, compounds **15** and **16** showed good binding affinity on COX-2 enzyme with free energies of binding, Δ*G* = −10.57 kcal/mol and −9.60 kcal/mol, respectively (Table 2). Halogen substitution appeared to have considerable importance but only flouro substitution affected the results in a positive way. When fluorine substitution is changed for chlorine or bromine atoms, the selectivity decreased (compound **14**). Interestingly, alkyl substitution also favored COX-2 selectivity. The best COX-2 selectivity was shown by compound **13**. For comparison, 3D representation of predicted binding mode and protein-ligand interactions of diflunisal in the active site of COX-2 enzyme is given in Figure 4. The 3D picture of compound **15** and **16** in the active site COX-2 enzymes are shown in Figure 5A,B. The COX-2 inhibitors were entering hydrophobic pocket consisted of Phe205, Phe381, Tyr348, Tyr385, Trp387 and Phe518. Other important interactions are halogen interactions, H-bonds interactions with His90, Glu198, Tyr355, Ser530 and Pi-Sulphur interactions with Met522 amino acid side chains, depending on the substituents of the phenyl group. The obtained computational modeling studies and binding energies support that the compounds **15** and **16** are potent and selective COX-2 inhibitors, agreeing with the experimental results.

## 3. Materials and Methods

### 3.1. General

All the chemicals were purchased from Merck (Darmstadt, Germany) or Sigma-Aldrich (St. Louis, MO, USA). Reactions were monitored by TLC on silica gel plates purchased from Merck. Melting points of the synthesized compounds were determined in a IA 9300 melting point apparatus (ThermoScientific, Staffordshire, UK) and are uncorrected. The purity of the compounds was verified by TLC, HPLC and elemental analysis. Elemental analysis was performed on a CHNS-932 apparatus (LECO, St. Joseph MI, USA). FT-IR spectra were recorded on a FT-IR-8400S spectrophotometer (Shimadzu, Columbia, MD, USA). NMR spectra were recorded on a Bruker spectrometer (Billerica, MA, USA) (300 MHz for ^1^H-NMR and 75 MHz for ^13^C-NMR, decoupled). Data are reported as follows: chemical shift, multiplicity (b.s.: broad singlet, d: doublet; m: multiplet, s: singlet, and t: triplet), coupling constants (Hz), integration. An Agilent 1100 LC-MS system (Agilent, Santa Clara, CA, USA) using the EI technique was employed for MS analysis. *Rf* × 100 values were obtained with petroleum ether/ethyl acetate (50:50 *v*/*v*) mobile phase at 21 °C. An Agilent 1100 series instrument equipped with a G1315A DAD detector, a pump (1311A Quat pump) and manual sampler was used for the identification of Rt (retention time) values; using a Zorbax SB C8 5 µm, 250 × 4.6 mm column. Operating conditions were performed by an isocratic HPLC grade acetonitrile/phosphate buffer (pH:3.7) (70:30 *v*/*v*) mobile phase at a flow rate of 1 mL/min. ChemStation A-08.03 (847)2000 software program with HP Compaq d330 DT computer was used to analyze the spectral data.

### 3.2. Synthesis of Methyl 2′,4′-Difluoro-4-hydroxybiphenyl-3-carboxylate (Diflunisal Ester, ***1***, CAS Number: 55544-00-8) and 2′,4′-Difluoro-4-hydroxybiphenyl-3-carbohydrazide (Diflunisal Hydrazide, ***2***)

These compounds were prepared and synthesized as described previously in our studies [10].

### 3.3. General Procedure for the Synthesis of 2-[(2′,4′-Difluoro-4-hydroxybiphenyl-3-yl)carbonyl]-N-(substituted)hydrazinocarbothioamides ***3***–***10***

Diflunisal hydrazide (**2**, 0.01 mol) was dissolved in ethanol (25 mL) and an equimolar amount of a substituted isothiocyanate was added to the reaction mixture which was refluxed for 2 to 6 h. The crude product was precipitated, filtered and washed with distilled water. The compounds were recrystallized from ethanol or DMF [12]. Compounds **3**, **4** and **5** were previously reported in our study [25].

*N-(3-Bromophenyl)-2-[(2′,4′-difluoro-4-hydroxybiphenyl-3-yl)carbonyl]hydrazine carbothioamide* (**6**): White solid. Yield 83%; m.p. 199 °C; MW: 478.309 g/mol; *Rf* × 100 value: 21; *Rt* value: 4.82 min. FT-IR ν max. (cm^−1^): 3244 (N-H), 1626 (Amide C=O), 1274 (C=S), 1141 (Ar-F). ^1^H-NMR (DMSO-*d*_6_/TMS) δ ppm: 7.09 (d, 1 H, Ar-H; *J* = 8.4 Hz), 7.22 (dt, 1H, Ar-H; *J* = 2.2 Hz), 7.24–7.40 (m, 3 H, Ar-H), 7.48–7.74 (m, 4 H, Ar-H), 8.06 (s, 1 H, Ar-H), 9.94 (s, 1 H, Ar-OH), 10.02 (s, 1 H, CSNH), 10.82 (s, 1 H, CONHNH), 12.01 (s, 1 H, CONH). ^13^C-NMR (DMSO-*d*_6_/TMS) δ ppm: 104.45 (C-9), 111.98 (C-11), 115.37 (C-5), 117.46 (C-3), 120.43 (C-17,18), 123.97 (C-7), 125.03 (C-1), 129.95 (C-13), 129.18 (C-14,16), 131.58 (C-12), 127.57 (C-2), 134.28 (C-6), 140.74 (C-15), 159.00 (C-4), 157.32–160.62 (C-8), 159.82–163.09 (C-10), 169.00 (C=O), 180.00 (C=S). HR-MS (EI, Calculated/Found *m*/*z*): 478.9937 (M++2), C_20_H_14_BrF_2_N_3_O_2_S 476.9952/476.9952 (M+), 383.0898/383.0902, 306.0295/306.0296, 264.0704/264.0700233.0403/233.0408. Anal. Cald. C_20_H_14_BrF_2_N_3_O_2_S Calculated/Found (%): C:50.22/50.22 H:2.95/3.27 N:8.79/8.77 S:6.70/6.60.

*N-(4-Bromophenyl)-2-[(2**′**,4**′**-difluoro-4-hydroxybiphenyl-3-yl)carbonyl]hydrazine carbothioamide* (**7**): White solid. Yield 93%; m.p. 196 °C; MW: 551.502 g/mol; *Rf* × 100 value: 62; *Rt* value: 5.02 min. FT-IR ν max. (cm^−1^): 3242 (N-H), 1637 (Amide C=O), 1238 (C=S), 1138 (Ar-F). ^1^H-NMR (DMSO-*d*_6_/TMS) δ ppm: 2.73 (s, 3 H, DMF methyl protons), 2.89 (s, 3 H, DMF(2) methyl protons), 7.08 (d, 1 H, Ar-H; *J* = 9 Hz), 7.21 (dt, 1 H, Ar-H; *J* = 1.8 Hz), 7.23 (dt, 1 H, Ar-H; *J* = 2.4 Hz), 7.35–7.63 (m, 6 H, Ar-H), 7.95 (s, 1 H, Ar-H), 8.07 (s, 1 H, CHO proton), 9.89 (s, 1 H, Ar-OH), 9.97 (s, 1 H, CSNH-Ar), 10.82 (s, 1 H, NHNHCS), 12.02 (s, 1 H, CONH). ^13^C-NMR (DMSO-*d*_6_/TMS) δ ppm: 104.45 (C-9), 111.98 (C-11), 115.35 (C-5), 117.44 (C-3), 123.97 (C-7), 125.01 (C-1), 127.64 (C-14,15,17,18), 129.18 (C-13), 130.32 (C-16), 131.58 (C-12), 134.27 (C-2), 138.51 (C-6), 158.87 (C-4), 157.35–160.65 (C-8), 159.88–163.09 (C-10), 167.00 (C=O), 180.00 (C=S). Anal. Cald. C_20_H_14_BrF_2_N_3_O_2_S.DMF Calculated/Found (%): C:50.10/50.03 H:3.84/3.96 N:10.16/10.12 S:5.82/5.98.

*N-({2-[(2′,4′-Difluoro-4-hydroxybiphenyl-3-yl)carbonyl]hydrazinyl}carbonothioyl)benzamide **(*****8**): White solid. Yield 91%; m.p. 233 °C; MW: 427.424 g/mol; *Rf* × 100 value: 16; *Rt* value: 4.90 min. FT-IR ν max. (cm^−1^): 3254 (N-H), 1676 (Benzoyl C=O) 1651 (Amide C=O), 1263 (C=S), 1139 (Ar-F). ^1^H-NMR (DMSO-*d*_6_/TMS) δ ppm: 7.13 (d, 1 H, Ar-H; *J* = 8.4 Hz), 7.19 (dd, 1 H, Ar-H; *J* = 6.6 Hz), 7.35 (dt, 1 H, Ar-H; *J* = 2.4 Hz), 7.52–7.69 (m, 6 H, Ar-H), 7.98 (d, 1 H, Ar-H; *J* = 7.2 Hz), 8.12 (t, 1 H, Ar-H; *J* = 2.1 Hz), 11.96 (s, 2 H, Ar-OH and CONHNH), 12.03 (s, 1 H, CSNH), 13.71 (s, 1 H, CONH). ^13^C-NMR (DMSO-*d*_6_/TMS) δ ppm: 104.48 (C-9), 112.08 (C-11), 115.74 (C-5), 117.32 (C-3), 123.82 (C-7), 125.71 (C-1), 128.50 (C-2), 129.69 (C-16), 130.15 (C-14,18), 130.39 (C-13), 131.48 (C-12), 131.73 (C-17,18), 134.20 (C-6), 140.74 (C-15), 159.75 (C-4), 157.32–160.62 (C-8), 159.96–163.09 (C-10), 168.45 (C=O), 171.50 (Benzoyl C=O), 180.00 (C=S). Anal. Cald. C_21_H_15_F_2_N_3_O_3_S Calculated/Found (%): C:59.01/58.38 H:3.54/3.98 N:9.83/9.61 S:7.50/7.27.

*2-[(2′,4′-Difluoro-4-hydroxybiphenyl-3-yl)carbonyl]-N-(2-methoxyphenyl)hydrazine carbothioamide* (**9**): White solid. Yield 82%; m.p. 195 °C; MW: 502.532 g/mol; *Rf* × 100 value: 68; *Rt* value: 4.48 min. FT-IR ν max. (cm^−1^): 3198 (N-H), 1645 (DMF C=O), 1633 (Amide C=O), 1240 (C=S), 1138 (Ar-F). ^1^H-NMR) (DMSO-*d*_6_/TMS) δ ppm: 2.73 (s, 3 H, DMF methyl protons), 2.88 (s, 3 H, DMF(2) methyl protons), 3.39 (water in solvent and O-CH_3_ protons), 6.95 (t, 1 H, Ar-H; *J* = 7.5 Hz), 7.08 (d, 1 H, Ar-H; *J* = 9 Hz), 7.16 (t, 1 H, Ar-H; *J* = 6.9 Hz), 7.21 (t, 1 H, Ar-H; *J* = 7.8 Hz), 7.36 (dt, 1 H, Ar-H; *J* = 2.4 Hz), 7.57–7.69 (m, 4 H, Ar-H), 7.95 (s, 1 H, Ar-H), 8.07 (s, 1 H, CHO protons), 9.36 (s, 1 H, Ar-OH), 9.84 (s, 1 H, CSNH-Ar), 10.85 (s, 1 H, NHNHCS), 11.44 (s, 1 H, thiol proton), 11.99 (s, 1 H, CONH). ^13^C-NMR (DMSO-*d*_6_/TMS) δ ppm: 55.63 (O-CH_3_), 104.38 (C-9), 111.44 (C-16), 112.00 (C-11), 115.83 (C-5), 117.32 (C-3), 119.79 (C-14), 123.94 (C-7), 125.17 (C-1), 126.12–126.72 (C-15,17,18), 127.51 (C-13), 131.55 (C-12), 129.42 (C-2), 134.01 (C-6), 159.50 (C-4), 157.31–160.58 (C-8), 159.78–162.06 (C-10), 166.20 (C=O), 180.00 (C=S). HR-MS (EI, Calculated/Found *m*/*z*): C_21_H_17_F_2_N_3_O_3_S 429.0953/429.0953, C_21_H_18_FN_3_O_2_S 395.1098/395.1098, C_13_H_10_F_2_N_2_O_2_ 264.0704/264.0704, C_13_H_7_F_2_O_2_ 233.0403/233.0408 Anal. Cald. C_21_H_17_F_2_N_3_O_3_S.DMF Calculated/Found (%): C:57.36/56.66 H:4.81/4.77 N:11.15/10.95 S:6.38/6.57.

*2-[(2′,4′-Difluoro-4-hydroxybiphenyl-3-yl)carbonyl]-N-(4-ethylphenyl)hydrazine carbothioamide* (**10**): White solid. Yield 89%; m.p. 185–186 °C; MW: 473.5454 g/mol; *Rf* × 100 value: 38; *Rt* value: 4.84 min. FT-IR ν max. (cm^−1^): 3234 (N-H), 1635 (Amide C=O), 1263 (C=S), 1139 (Ar-F). ^1^H-NMR) (DMSO-*d*_6_/TMS) δ ppm: 1.05 (t, ethanol CH_3_), 1.17 (t, 3 H, C_6_H_4_-CH_2_-CH_3_ *J* = 7.5 Hz), 2.57 (q, 2 H, C_6_H_4_-CH_2_-CH_3_ *J* = 7.5 Hz), 3.40 (water in solvent and Ethanol CH_2_), 4.39 (s, Ethanol OH), 7.06 (d, 1 H, Ar-H; *J* = 8.7 Hz), 7.15–7.25 (m, 2 H, Ar-H), 7.32–7.40 (m, 2 H, Ar-H), 7.55–7.64 (m, 4 H, Ar-H), 8.06 (s, 1 H, Ar-H), 9.82 (s, 2 H, Ar-OH ve CSNH-Ar), 10.85 (s, 1 H, NHNHCS), 12.04 (s, 1 H, CONH). ^13^C-NMR) (DMSO-*d*_6_/TMS) δ ppm: 15.55–27.61 (CH_2_CH_3_) 104.38 (C-9), 111.93 (C-11), 115.59 (C-5), 117.38 (C-3), 123.96 (C-7), 125.04 (C-1), 127.44 (C-14,18), 128.03 (C-15,17), 129.19 (C-13), 131.56 (C-12), 134.14 (C-2), 136.55 (C-6), 139.17 (C-16), 158.50 (C-4), 157.32–160.60 (C-8), 159.81–163.07 (C-10), 168.00 (C=O), 180.00 (C=S). Anal. Cald. C_22_H_19_F_2_N_3_O_2_S. C_2_H_5_OH Calculated/Found (%): C:60.81/60.24 H:5.27/5.23 N:8.86/8.91 S:6.75/6.87.

### 3.4. General Procedure for the Synthesis of 5-(2′,4′-Difluoro-4-hydroxybiphenyl-3-yl) -4-(substituted)-2,4-dihydro-3H-1,2,4-triazole-3-thiones ***11***–***16***

Compounds **3**–**9** except for compound **8** and **10** (0.01 mol) were dissolved in sodium hydroxide solution (2 N, 25 mL) and heated under reflux for 4 to 6 h. The solution then was neutralized with concentrated hydrochloric acid. The crude product was precipitated, filtered and washed with distilled water. The compounds recrystallized twice from ethanol or DMF [12].

*5-(2′,4′-Difluoro-4-hydroxybiphenyl-3-yl)-4-propyl-2,4-dihydro-3H-1,2,4-triazole-3-thione* (**11**): White solid. Yield 69%; m.p. 177–179 °C; MW: 347.3823 g/mol; *Rf* × 100 value: 35; *Rt* value: 3.81 min. FT-IR ν max. (cm^−1^): 3190 (N-H), 1626 (triazole C=N), 1257 (C=S), 1141 (Ar-F). ^1^H-NMR (DMSO-*d*_6_/TMS) δ ppm: 0.66 (t, 3 H, CH_2_-CH_2_-CH_3_ *J* = 7.3 Hz), 1.54 (m, 2 H, CH_2_-CH_2_-CH_3_), 3.84 (t, 2 H, CH_2_-CH_2_-CH_3_ *J* = 7.3 Hz), 7.12 (d, 1 H, Ar-H; *J* = 8.7 Hz), 7.17 (dd, 1 H, Ar-H; *J* = 2.1 Hz), 7.32 (dt, 1 H, Ar-H; *J* = 2.4 Hz), 7.47–7.61 (m, 3 H, Ar-H), 13.76 (*bs*, 1 H, triazole NH). AR-OH exchanged with deuterium of solvent. Anal. Cald. C_17_H_15_F_2_N_3_OS Calculated/Found (%): C:58.78/58.28 H:4.35/4.50 N:12.10/11.99 S:9.23/9.56.

*5-(2′,4′-Difluoro-4-hydroxybiphenyl-3-yl)-4-(3-fluorophenyl)-2,4-dihydro-3H-1,2,4-triazole-3-thione* (**12**): White solid. Yield 98%; m.p. 244 °C; MW: 399.3889 g/mol; *Rf* × 100 value: 37; *Rt* value: 3.81 min. FT-IR ν max. (cm^−1^): 3250 (N-H), 1633 (triazole C=N), 1255 (C=S), 1139 (Ar-F). ^1^H-NMR (DMSO-*d*_6_/TMS) δ ppm: 6.84 (d, 1 H, Ar-H; *J* = 8.7 Hz), 7.12–7.18 (m, 2 H, Ar-H), 7.19–7.31 (m, 2 H, Ar-H), 7.32–7.53 (m, 5 H, Ar-H), 10.32 (s, 1 H, Ar-OH), 14.14 (*bs*, 1 H, triazole NH). ^13^C-NMR) (DMSO-*d*_6_/TMS) δ ppm: 104.43 (C-9), 111.94 (C-11), 113.27 (C-5), 115.46 (C-17) 115.75 (C-16), 115.99 (C-3), 123.77 (C-7), 124.20 (C-14), 124.75 (C-1), 130.17 (C-13), 132.54 (C-2), 131.36 (C-12), 131.73 (C-18), 135.77 (C-6), 149.20 (C-4), 155.62 (C=N), 157.22–160.57 (C-8), 159.71–162.97 (C-10), 159.62 (C-15), 167.68 (C=S). Anal. Cald. C_20_H_12_F_3_N_3_OS Calculated/Found (%): C:60.15/59.85 H:3.03/3.27 N:10.52/10.46 S:8.03/8.16.

*4-(3-Chlorophenyl)-5-(2′,4′-difluoro-4-hydroxybiphenyl-3-yl)-2,4-dihydro-3H-1,2,4-triazole-3-thione* (**13**): White solid. Yield 95%; m.p. 243–244 °C; MW: 415.8435 g/mol; *Rf* × 100 value: 36; *Rt* value: 3.94 min. FT-IR ν max.(cm^−1^): 3188 (N-H), 1620 (triazole C=N); 1269 (C=S), 1139 (Ar-F). ^1^H-NMR (300 MHz) (DMSO-*d*_6_/TMS) δ ppm: 6.84 (d, 1 H, Ar-H; *J* = 8.4 Hz), 7.15 (dt, 1 H, Ar-H; *J* = 2.1 Hz), 7.24–7.31 (m, 1 H, Ar-H), 7.34 (t, 1 H, Ar-H; *J* = 2.2 Hz), 7.35 (d, 1 H, Ar-H; *J* = 2.7 Hz), 7.39–7.56 (m, 4 H, Ar-H), 7.57 (s, 1 H, Ar-H), 10.34 (s, 1 H, Ar-OH), 14.16 (*bs*, 1 H, triazole NH). ^13^C-NMR (DMSO-*d*_6_/TMS) δ ppm: 104.44 (C-9), 111.93 (C-11), 113.22 (C-5), 115.99 (C-3), 123.77 (C-7), 124.80 (C-1), 126.66–128.14–128.89 (C-14,16,17), 130.18 (C-13), 132.45 (C-2), 131.38 (C-12), 131.81 (C-18), 132.65 (C-15), 135.62 (C-6), 149.19 (C-4), 155.54 (C=N), 157.24–160.53 (C-8), 159.71–162.98 (C-10), 167.63 (C=S). Anal. Cald. C_20_H_12_ClF_2_N_3_OS Calculated/Found (%): C:57.77/57.54 H:2.91/3.25 N:10.10/10.13 S:7.71/8.03.

*4-(3-Bromophenyl)-5-(2′,4′-difluoro-4-hydroxybiphenyl-3-yl)-2,4-dihydro-3H-1,2,4-triazole-3-thione* (**14**): White solid. Yield 84%; m.p. 232–233 °C; MW: 460.2945 g/mol; *Rf* × 100 value: 36; *Rt* value: 4.18 min. FT-IR ν max.(cm^−1^): 3201 (N-H), 1620 (triazole C=N), 1240 (C=S), 1139 (Ar-F). ^1^H-NMR (300 MHz) (DMSO-*d*_6_/TMS) δ ppm: 6.85 (d, 1 H, Ar-H; *J* = 8.7 Hz), 7.14 (dt, 1 H, Ar-H; *J* = 2.1 Hz), 7.28–7.59 (m, 8 H, Ar-H), 10.42 (s, 1 H, Ar-OH), 14.12 (*bs*, 1 H, triazole NH). ^13^C-NMR (DMSO-*d*_6_/TMS) δ ppm: 104.43 (C-9), 111.93 (C-11), 113.19 (C-5), 116.05 (C-3), 123.81 (C-7), 124.69 (C-1), 127.09 (C-14,16), 130.44 (C-13), 130.96 (C-17,18), 131.36 (C-12), 131.68 (C-15), 132.46 (C-2), 135.91 (C-6), 149.21 (C-4), 155.69 (C=N), 157.22–160.52 (C-8), 159.69–162.95 (C-10), 167.59 (C=S). HR-MS (EI, Calculated/Found *m*/*z*): 460.9822 (M+2), C_20_H_12_BrF_2_N_3_OS 458.9846/458.9847 (M+), C_20_H_12_BrF_2_N_3_O 427.0126/427.0126, 347.0853/347.0856, 231.0490/231.0492. Anal. Cald. C_20_H_12_BrF_2_N_3_OS Calculated/Found (%): C:52.19/51.31 H:2.63/3.01 N:9.13/9.06 S:6.97/6.62.

*4-(4-Bromophenyl)-5-(2′,4′-difluoro-4-hydroxybiphenyl-3-yl)-2,4-dihydro-3H-1,2,4-triazole-3-thione* (**15**): White solid. Yield 69%; m.p. 263–264 °C; MW: 460.294 g/mol; *Rf* × 100 value: 60; *Rt* value: 4.38 min. FT-IR ν max. (cm^−1^): 3196 (N-H), 1622 (triazole C=N), 1242 (C=S), 1139 (Ar-F). ^1^H-NMR (DMSO-*d*_6_/TMS) δ ppm: 6.84 (d, 1 H, Ar-H; *J* = 8.7 Hz), 7.14 (dt, 1 H, Ar-H; *J* = 2.4 Hz), 7.28 (d, 2 H, Ar-H, o-Br, *J* = 6.6 Hz), 7.29–7.35 (m, 2 H, Ar-H), 7.43–7.60 (m, 2 H, Ar-H), 7.61 (d, 2 H, Ar-H, m-Br, *J* = 6.6 Hz), 10.27 (s, 1 H, Ar-OH), 14.13 (s, 1 H, NH). ^13^C-NMR (DMSO-*d*_6_/TMS) δ ppm: 104.45 (C-9), 111.90 (C-11), 113.30 (C-5), 116.01 (C-3), 121.95 (C-14,18), 123.75 (C-7), 124.82 (C-1), 130.02 (C-15,17), 131.40 (C-12), 131.57 (C-13), 131.80 (C-16), 132.62 (C-2), 133.76 (C-6), 149.25 (C-4), 155.47 (C=N), 157.24–160.53 (C-8), 159.70–162.97 (C-10), 167.58 (C=S). Anal. Cald. C_20_H_12_BrF_2_N_3_OS Calculated/Found (%): C:52.19/52.27 H:2.63/2.91 N:9.13/8.80 S:6.97/6.77.

*5-(2′,4′-Difluoro-4-hydroxybiphenyl-3-yl)-4-(2-methoxyphenyl)-2,4-dihydro-3H-1,2,4-triazole-3-thione* (**16**): White solid. Yield 65%; m.p. 196 °C; MW: 457.493 g/mol; *Rf* × 100 value: 60; *Rt* value: 3.90 min. FT-IR ν max. (cm^−1^): 3198 (N-H), 1626 (triazole C=N), 1257 (C=S), 1141 (Ar-F). ^1^H-NMR (DMSO-d6/TMS) δ ppm: 1.05 (ethanol CH_3_), 3.43 (Ethanol CH_2_), 3.55 (s, 3 H, Ar-O-CH_3_), 3.90 (ethanol OH), 6.85 (d, 1 H, Ar-H; *J* = 8.4 Hz), 6.80–7.04 (m, 2 H, Ar-H), 7.13 (dt, 1 H, Ar-H; *J* = 2.1 Hz), 7.25–7.39 (m, 6 H, Ar-H), 10.31 (s, 1 H, Ar-OH), 14.02 (*bs*, 1 H, NH). ^13^C-NMR (DMSO-*d*_6_/TMS) δ ppm: 55.41 (O-CH_3_), 104.42 (C-9), 111.93 (C-11), 112.28 (C-15), 116.02 (C-5), 120.14 (C-17) 122.68 (C-16,18), 123.80 (C-7), 124.39 (C-1), 130.34 (C-13), 130.80 (C-2), 131.23 (C-12), 132.14 (C-6), 133.50 (C-3), 149.89 (C-4), 154.21 (C-14), 155.75 (C=N), 157.19–160.48 (C-8), 159.64–162.90 (C-10), 168.19 (C=S). HR-MS (EI, Calculated/Found *m*/*z*): C_21_H_15_F_2_N_3_O_2_S 411.0847/411.0847 (M+), C_21_H_14_F_2_N_3_O_2_ 378.1043/378.1048, 231.0490/231.0478. Anal. Cald. C_21_H_15_F_2_N_3_O_2_S. C_2_H_5_OH Calculated/Found (%): C:60.32/60.06 H:4.59/4.05 N:9.18/9.63 S:6.99/7.26.

### 3.5. Cell Culture Conditions

In this project, the colorectal cancer cell line HCT116 (ATCC^®^ CCL-247™), androgen-dependent prostate cancer cell line PC-3 (ATCC^®^ CRL-1435™), breast cancer cell lines T47D (ATCC^®^ HTB-133™), MCF7 (ATCC^®^ HTB-22™) and finally, epithelial embryonic kidney cell line HEK293 (ATCC^®^ CRL-1573™) were used. All media were completed with %10 fetal bovine serum (FBS) and %1 penicillin streptomycin amphotericin (PSA). All cells were cultured periodically at 37 °C, 5% (*v*/*v*) CO_2_ and 95% (*v*/*v*) air in T-75 flasks. Different media were used for the different cell lines based on ATCC standards: MCF7 and HEK293 cell lines were cultured in DMEM High Mmedium (ATCC, 30-2003); T47D and PC-3 cell lines were cultured in RPM 1640 medium (ATCC, 30-2001); HCT116 cell line was cultured in DMEM F12 medium (ATCC, 30-2006). RPM 1640 medium was further completed with 0.2 unit/mL bovine insulin for the T47D cell line. Cells were passaged when they reached %80 confluency in order to support proliferation.

#### 3.5.1. Cytotoxicity Assay and Determination of IC_50_ Values of Synthesized Compounds

The effects of the synthesized compounds on the cell viability were tested. Stock solutions for each compound were prepared by measuring 20 mg of each compound and dissolving it in 1 mL DMSO to a final concentration of 20 mg/10 mL, followed by filtration through a 0.2-μm filter (Sartorius AG, Gottingen, Germany). Then stock solutions were diluted as 1/10, 1/100 and 1/1000 in the designated media and applied on the cell lines. In order to determine the effect of DMSO itself, the same dilutions were performed by using DMSO, only and applied on the cell lines. Cell densities used for each cell line in 96 well plate were as follows: for PC-3, HCT116 and HEK293 cell lines 5000 cells/well; for T47D cell lines 20,000 cells/well. Cell viability was measured by the MTS cytotoxicity assay (CellTiter96 AqueousOne Solution; Promega, Southampton, UK) according to the manufacturer’s instructions. 3-(4,5-Dimethylthiazol-2-yl)-5-(3-carboxymethoxyphenyl)-2-(4-sulfo-phenyl)-2*H*-tetrazolium (MTS) is a yellow tetrazolium salt which is catabolized to formazan by dehydrogenase enzyme in the mitochondria of the living cells. Formazan is a purple compound and the detection in this assay is based on the measurement of formazan compounds by an ELISA plate reader. After incubating the cells for 72 h, 10 µL MTS reagent and 100 µL designated medium was given to the cells followed by 2 h of incubation at 37 °C. Thereafter, the absorbance at 490 nm was measured by an ELISA plate reader. (BioTek Instruments, Inc., Winooski, VT, USA). The activity doses of the compounds were determined according to 50% cell death rate when compared to DMSO, only after 72 h of incubation. Compounds that do not affect cell viability of healthy cell line (HEK293) but deathly to cancer cell lines were further tested with narrower test conditions for MTS assay to determine IC_50_ values. IC_50_ values are calculated by the GraphPad prism software.

#### 3.5.2. Annexin V

Determination of IC_50_ values was followed by the investigation of effect of the synthesized compounds on apoptosis. Cells were seeded into six well plates at densities of 15,000 cells/well (PC-3, HCT116 and HEK293 cell lines) and 60,000 cells/well (T47D cell lines). After the treatment cells were harvested and washed with ice cold PBS. 1 × Annexin-binding buffer was prepared while the cells were being washed. Working solution of PI (100 µg/mL) was prepared as well by diluting 5 µL of the 1 mg/mL PI stock solution in 45 µL 1 × Annexin-binding buffer. After the precipitation of the cells, they were resuspended in 500 µL 1 × Annexin-binding buffer. Resuspended cells were separated into four groups (Annexin V, PI, Annexin V + PI and NC) each containing 100 µL of suspension. Five µL of the Annexin V conjugate was added to the proper groups. Samples were incubated for 15 min at room temperature. The incubation step was followed by adding 2 µL of the 100 µg/mL PI working solution to the proper groups then the fluorescence was observed by flow cytometry.

#### 3.5.3. 5-Ethynyl-2′-deoxyuridine (EdU) Staining

The EdU staining protocol directly measures the level of active DNA synthesis of the cell cycle. This measurement enables one to see dividing cells under a fluorescence microscope. In order to perform EdU staining cells were seeded on the coverslips which were located in the wells of six well plates. As in the Annexin V procedure cells were seeded into six well plates at densities of 15,000 cells/well (PC-3, HCT116 and HEK293 cell lines) and 60,000 cells/well (T47D cell lines). After drug treatment, cells that are on the coverslips were incubated with 10 µM EdU. The incubation step is followed by cell fixation and permeabilization. Fixation occurred with 3.7% formaldehyde in PBS for 15 min then cells were washed with 3% BSA in PBS. Permeabilization was performed by incubating cells in 0.5% Triton^®^ X-100 in PBS for 20 min at room temperature. Click-iT^®^ reaction cocktail (0.5 mL) was added to the cells after washing with 3% BSA solution. Cells were incubated for 30 min at room temperature, then DNA synthesis was observed under a confocal microscope.

### 3.6. Docking Studies

The preparation of the protein, as well as the compounds were performed in BIOVIA Discovery Studio 4.5. The crystal structures of the enzymes COX-1 and COX-2 were downloaded from the PDB database (accession code: 1EQG for COX-1 [46] and 3NT1 for COX-2 [47] (http://www.rcsb.org/pdb/). All protein structures were cleaned of inhibitors, water molecules and non-interacting ions, while cofactors were retained. All hydrogens were added and proteins minimized using the “Clean Geometry” toolkit. For more complete optimization, proteins were submitted to the “Prepare Macromolecule” protocol. Missing hydrogen atoms were added based on the protonation state of the titratable residues at pH 7.4. Compounds were prepared and minimized in the same force field. After preparation, docking was performed in AutoDock 4.2 program (http://autodock.scripps.edu). All the compounds were set to be flexible, while the proteins were kept rigid. AutoDock adds a free-energy scoring function created from a linear regression analysis, the AMBER force field, and a large set of diverse protein-ligand complexes with known inhibition constants. The coordinates of alpha carbon of Tyr355 were used for grid-centering for COX enzymes, grid box was set to be 70 grid points (each grid point is 0.375 Å). For the docking studies the Lamarkian genetic algorithm was used as search algorithm. For visualization of non-bonded interactions, we also used BIOVIA Discovery Studio 4.5.

## 4. Conclusions

Diflunisal and its derivatives have been widely studied and different biological activities have been reported for these compounds. The correlation between the COX-2 selectivity and anticancer activity, had become the basic subject of our research. Thiosemicarbazides and 1,2,4-triazole-3-thiones have been related with anticancer activity, therefore, we have designed some novel compounds combining these active pharmacophores and a NSAID.

In this study, we have synthesized new diflunisal thiosemicarbazides and 1,2,4-triazole-3-thiones in order to determine their possible anticancer activity. The synthesis, structure characterization and antibacterial activity against *Helicobacter pylori* of thiosemicarbazides, which are the prototypes of the 1,2,4-triazoles **11**–**13**, are given previously study [25]. Compounds **6** and **10** were found active against the PC-3 cancer cell line, with IC_50_ values of 41.8 and 11.7 µM, respectively. Compound **16** was found active against the HCT-116 cancer cell line with a 6.2 µM IC_50_ value. Compounds **15** and **16** were found active against the T47D cancer cell line with IC_50_ values of 43.4 and 27.3 µM, respectively. The in silico and in vitro studies that we performed lead us to the conclusion that there is a correlation between COX-2 enzyme activity and anticancer activity in vitro. In the docking studies, compounds **12** and **13** appeared to show the most affinity towards COX-2 enzyme however, compounds **15** (Δ*G* = −10.11 kcal/mol) and **16** (Δ*G* = −9.41 kcal/mol) showed high inhibition on COX-2 enzyme. The highest selectivity towards COX-2 was shown by compound **13**. Further experiments are still needed in order to improve our understanding of how these compounds elicit their anticancer activity.

## Figures and Tables

**Table 1:** IC_50_ values (µM) of compounds.

Compound	PC-3	HCT116	T47D	MCF7	HEK
**3**	ND	ND	ND	ND	ND
**4**	PCV	PCV	PCV	PCV	617 + 35
**5**	67,2 ± 4.5	PCV	28,2 ± 2.5	PCV	44,4 ± 1,7
**6**	41.8 ± 3.4	PCV	100 ± 2.3	PCV	93 ± 3.43
**7**	11.7 ± 4.2	PCV	16.73 ± 2.8	PCV	3.17 ± 1.06
**8**	2.85 ± 3.6	3.68 ± 2.1	8.07 ± 2.0	PCV	5.04 ± 1.5
**9**	1.575 ± 1.4	1.83 ± 0.92	3.86 ± 0.7	3.92 ± 0.27	3.02 ± 1.9
**10**	11.7 ± 2.6	PCV	NSC	PCV	94 ± 3.74
**11**	174.13 ± 6.3	NSC	10.05 ± 1.57	22.18 ± 2.3	11.52 ± 0.2
**12**	33.95 ± 2.1	NSC	9.24 ± 1.01	15.03 ± 1.3	10.66 ± 0.96
**13**	34.09 ± 3.7	NSC	5.55 ± 2.7	12.883 ± 2.1	8.7 ± 1.79
**14**	PCV	PCV	11.47 ± 1.8	NSC	12,8 ± 2.1
**15**	145 ± 2.8	PCV	43.4 ± 2.72	PCV	145 ± 2.3
**16**	168.86 ± 3.9	6.2 ± 3.1	27.3 ± 2.28	PCV	53 ± 2.39
Cisplatin	* 39.9 µM	* 1.25 µM	* 0.836 µM		

PCV: Promoting cell viability; NSC: No significant change; ND: Not detected, experiment could not be performed, compound was precipitated; * Lit [42,43].

**Table 2:** Docking study results of synthesized compounds.

Compound	COX-1	COX-2
**13**	Δ*G* = −10.07 kcal/mol	Δ*G* = −11.20 kcal/mol
**14**	Δ*G* = −10.33 kcal/mol	Δ*G* = −10.66 kcal/mol
**15**	Δ*G* = −10.11 kcal/mol	Δ*G* = −10.57 kcal/mol
**16**	Δ*G* = −9.41 kcal/mol	Δ*G* = −9.60 kcal/mol
